# Res Ipsa - Testicular histology after feminizing hormone therapy

**DOI:** 10.4322/acr.2021.336

**Published:** 2021-11-12

**Authors:** Ameer Hamza

**Affiliations:** 1 University of Kansas Medical Center, Department of Pathology and Laboratory Medicine, Kansas City, Kansas, USA

**Keywords:** Gender Dysphoria, Gender Identity, Sex Reassignment Surgery

Pathology community is aware of the “hot seat” or unknown slide conference. For the author during his residency training the “hottest” seat was the “Res Ipsa” cases during unknown slide conference by one of the faculty members. Res Ipsa (Latin for “the thing speaks for itself”) was “all” that was provided as the clinical history / relevant information needed to diagnose the case. Thanks to A&CR for providing a platform for my Res Ipsa. Thankfully, I am not the one on the hot seat today.

Hormonal therapy followed by gender reassignment surgery is now the standard of care in people with gender dysphoria. Clinical Practice Guideline from endocrine societies in fact recommend gender-affirming surgery only after completion of at least 1 year of consistent and compliant hormone treatment.^1^

The objective of hormonal therapy is to suppress endogenous hormone levels and replace them with exogenous hormones of the desired sex. The hormone therapy regimen for male-to-female gender transition involves the use of an antiandrogen in conjunction with an estrogen. The antiandrogens demonstrate their effect by reducing endogenous testosterone levels to those found in adult biological female. This also enables the estrogen therapy to have its maximal effect.

Orchiectomy is one of the surgeries patients seeking male-to-female physical adaptation must undergo. Depending upon institutional policy these specimens are usually subjected to histopathologic evaluation.

Histopathologic alterations are mostly hormone therapy related changes and they are present in almost all cases, albeit to variable extent. Some of the most common changes as described in the literature include reduced diameter of seminiferous tubules and peri-tubular fibrosis, aspermatogenesis with maturation arrest, and absence of Leydig cells.^2^ Less common findings include hyperplasia of epididymal epithelium and peri-epididymal fibrosis.^2^ Despite the presence of these changes in virtually all the cases the gross appearance as well as the weight of the specimens is usually within the normal range.^2^^,^^3^

Most histologic changes in the testicular parenchyma including reduced / absent spermatogenesis are secondary to alterations in the physiologic mechanisms governed by testosterone as well as estrogenization. Additionally, as the epididymal epithelium is shown to express ER,^2^ the epididymal hyperplasia identified in some cases can also be attributed to exogenous estrogens. It is, however, unclear whether the changes are related to demographic factors, or if they are dependent on estrogen regimen and timing of exposure to estrogen i.e. pre-pubertal versus post-pubertal.

Pathologists usually find themselves struggling to find appropriate wording for signing out such cases. The following example may be helpful in this regard.

- Right and left testes, bilateral orchiectomy:
  Testicular parenchyma with fibrosis of the seminiferous tubules, hyalinization surrounding seminiferous tubules, reduced / absent spermatogenesis, and reduced / absent Leydig cells, consistent with hormone therapy related changes.

**Figure 1:**
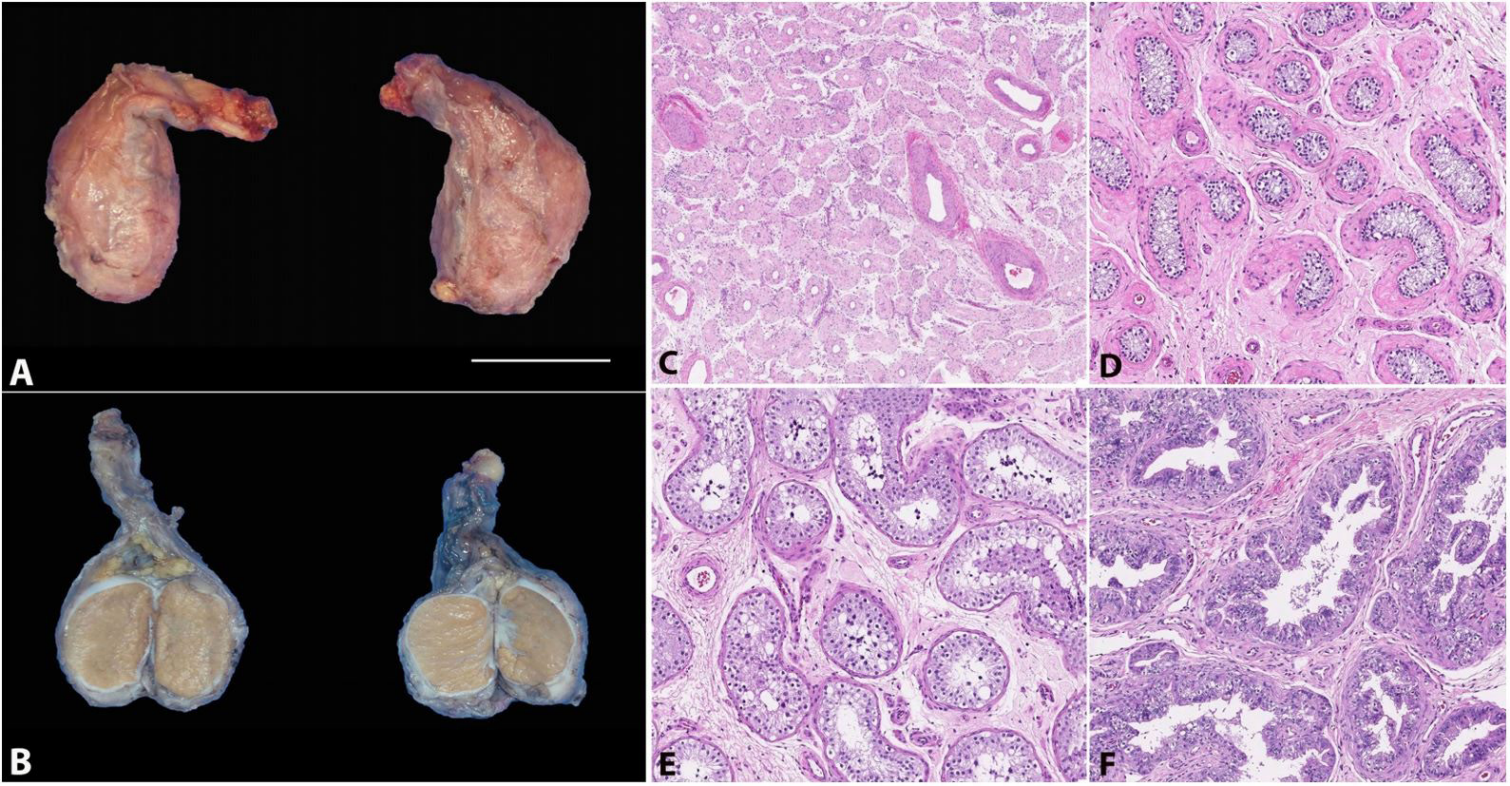
**A** and **B** demonstrate the gross appearance and cut surface of the specimen (scale bar = 3 cm). Figures **C**-**F** show the common histologic findings including fibrosis of seminiferous tubules and absent Leydig cells (**C**; H&E 40X), hyalinization / fibrosis surrounding the seminiferous tubules (**D**; H&E 100X), reduced spermatogenesis with maturation arrest (**E**; H&E 100X), peri-epididymal fibrosis and hyperplasia of epididymal epithelium (**F**; H&E 100X).

The images depict an orchiectomy specimen from a 24-year-old adult who underwent gender reassignment surgery after hormonal therapy with estradiol, micronized progesterone, and spironolactone. Figure 1A and 1B demonstrate the gross appearance and cut surface of the specimen, showing no alteration in the gross appearance. The images depict true size (1X). Figure 1C to 1F demonstrate some of the histologic findings in these specimens including fibrosis of seminiferous tubules and absent Leydig cells (1C; H&E 40X), hyalinization surrounding the seminiferous tubules (1D; H&E 100X), reduced spermatogenesis with maturation arrest (1E; H&E 100X), peri-epididymal fibrosis and hyperplasia of epididymal epithelium (1F; H&E 100X).

The “Res Ipsa” in this case are the hormone therapy related changes that point one towards the fact that this is an orchiectomy specimen from a patient undergoing gender reassignment surgery.
